# Quality of service and continuous quality improvement in voluntary medical male circumcision programme across four provinces in South Africa: Longitudinal and cross-sectional programme data

**DOI:** 10.1371/journal.pone.0254850

**Published:** 2021-08-05

**Authors:** Tawanda Nyengerai, Motshana Phohole, Nelson Iqaba, Constance Wose Kinge, Elizabeth Gori, Khumbulani Moyo, Charles Chasela

**Affiliations:** 1 Department of Epidemiology and Biostatistics, School of Public Health, Faculty of Health Sciences, University of the Witwatersrand, Johannesburg, Gauteng, South Africa; 2 Voluntary Medical Male Circumcision (VMMC) Programme, Right to Care, Johannesburg, Gauteng, South Africa; 3 Department of Implementation Science, Right to Care, Johannesburg, Gauteng, South Africa; 4 Department of Pre-Clinical Veterinary Science, University of Zimbabwe, Harare, Zimbabwe; University of Washington, UNITED STATES

## Abstract

**Background:**

Recent studies in the Sub-Saharan countries in Africa have indicated gaps and challenges for voluntary medical male circumcision (VMMC) quality of service. Less has focused on the changes in quality of service after implementation of continuous quality improvement (CQI) action plans. This study aimed to evaluate the impact of coaching, provision of standard operating procedures (SOPS) and guidelines, mentoring and on-site in-service training in improving quality of VMMC services across four Right to Care (RTC) supported provinces in South Africa.

**Method:**

This was a pre- and post-interventional study on RTC supported VMMC sites from July 2018 to October 2019. All RTC-supported sites that were assessed at baseline and post-intervention were included in the study. Data for baseline CQI assessment and re-assessments was collected using a standardized National Department of Health (NDoH) CQI assessment tool for VMMC services from routine RTC facility level VMMC programme data. Quality improvement support was provided through a combination of coaching, provision of standard operating procedures and guidelines, mentoring and on-site in-service training on quality improvement planning and implementation. The main outcome measure was quality of service. A paired sample t-test was used to compare the difference in mean quality of service scores before and after CQI implementation by quality standard.

**Results:**

A total of 40 health facilities were assessed at both baseline and after CQI support visits. Results showed significant increases for the overall changes in quality of service after CQI support intervention of 12% for infection prevention (95%CI: 7–17; p<0.001) and 8% for male circumcision surgical procedure, (95%CI: 3–13; p<0.01). Similarly, individual counselling, and HIV testing increased by 14%, (95%CI: 7–20; p<0.001), group counselling, registration and communication by 8%, (95%CI: 3–14; p<0.001), and 35% for monitoring and evaluation, (95%CI: 28–42; p<0.001). In addition, there were significant increases for management systems of 29%, (95%CI: 22–35; p<0.001), leadership and planning 23%, (95%CI: 13–34; p<0.001%) and supplies, equipment, environment and emergency 5%, (95%CI: 1–9; p<0.01). The overall quality of service performance across provinces increased by 18% (95%CI: 14–21; p<0.001).

**Conclusion:**

The overall quality of service performance across provinces was significantly improved after implementation of CQI support intervention program. Regular visits and intensive CQI support are required for sites that will be performing below quality standards.

## Introduction

Clinical trials that were conducted in Kenya, Uganda and South Africa have provided evidence that medical male circumcision (MC) reduces the risk of female-to-male sexual transmission of HIV by approximately 60% [1, 2] In addition to the reduction in HIV transmission risk, VMMC minimises the risk of other sexually transmitted infections (STIs) and this include herpes and syphilis [3]. Furthermore, research findings have substantiated that VMMC saves costs through the prevention of new HIV infections and by minimising the proportion of people on highly active antiretroviral treatment (HAART) [4]. Mathematical modelling studies have also demonstrated that more than 3 million HIV infections can be averted by 2025 if VMMC coverage is maintained above 80% [2, 4–6].

In line with these positive developments in research, countries with high HIV prevalence were encouraged by World Health Organisation (WHO) and the United Nations Programme on HIV and AIDS (UNAIDS) to use VMMC as an additional strategy for HIV prevention [1, 4, 7]. South Africa introduced VMMC as one of the prevention methods for HIV transmission in 2010 [8]. In 2003, the highest rates of medical MC were in the Western Cape and lowest in Gauteng [9]. In 2018, more than 572,000 circumcisions were performed, compared to 485,500 in 2015 [8]. This is evidence of positive developments in the scaling up of VMMC programmes in South Africa.

However, scaling up of VMMC may compromise the quality of service delivery [10, 11]. Poor quality of service can result in increased cost of operations, negative health outcomes, adverse events (AEs), customer dissatisfaction and negative word of mouth [12]. Research also indicate that communication, medical staff skills and availability of resources such as equipment and supplies can affect the attitudes and perceptions of clients on the quality of service offered [12].

Rate of AEs in VMMC programs is among measurement tools used to assess quality of service and they affect the desire for individuals to undergo medical MC [13, 14]. Fear of pain, medical complications and the possibility of other negative outcomes from VMMC program act as a barrier for demand creation [15]. Poor monitoring and management of AEs have a substantial negative impact on VMMC service delivery expectations [16, 17]. Findings from previous studies showed that the majority of AEs were procedure related [18]. Young age groups mostly experience moderate to severe AEs that are related to surgical procedures compared to prepex method [19, 20]. Evidence also indicate that the majority of AEs were associated with infections and this frequently affected young age groups [13, 20, 21]. Device displacement and bleeding are the most commonly reported AEs for VMMC programs [13]. It is important to identify factors which contribute to young age groups being more susceptible to AEs related to surgical procedures.

The WHO, UNAIDS and the United States President’s Emergency Plan for AIDS Relief (PEPFAR) have created a list of core essential elements as a set of minimum quality standards to address quality of service gaps for VMMC programmes [2, 22, 23]. These elements include; individual counselling and HIV Testing for VMMC Clients (8 Sub-standards), management systems (8 Sub-standards), MC surgical procedure (9 Sub-standards), supplies, equipment and environment (8 Sub-standards), monitoring and evaluation (4 Sub-standards), registration, group education and IEC (4 Sub-standards), infection prevention (14 Sub-standards), leadership and planning (2 Sub-standards) [22, 24, 25].

However, 86% of the WHO VMMC priority countries were operating with inadequate supplies and equipment [26]. In South Africa, a significant proportion of sites were operating with inadequate VMMC supplies and equipment, and also without guideline documents [11]. This has a negative impact for VMMC quality of service. Findings from previous studies also shows that 72% of VMMC facilities in South Africa were operating without the required basic life support equipment [11]. Furthermore, the South Africa 2016 reference report on HIV and TB investment case showed inefficiencies in the supply chain for the healthcare sector in South Africa and the Eastern Cape was the most seriously affected province [27]. Further declines in quality of service were also reported in South Africa for counselling, infection control, monitoring and evaluation [4, 28–30].

Hence it is important to adopt continuous quality improvement (CQI) processes to identify challenges and quality of service gaps. CQI processes helps to determine the level of quality improvement support required, and also to measure the changes in quality of service after CQI support. Evidence from previous studies indicates that CQI principles, strategies and techniques are critical drivers of new quality of service models [31]. Implementation and support for CQI activities involves creating site level quality improvement teams that will focus on addressing quality of service gaps. Literature shows that facilities with functional CQI teams, demonstrated faster improvement compared to facilities without quality improvement teams [26, 32]. A quality improvement framework with roles and responsibilities for facility-based CQI teams helps in the delivery of positive quality improvement outcomes [33].

However, less has been focused on the changes in quality of services across all the quality standards after CQI support. Quality of service has a bearing on the willingness of males to undergo medical MC. MC procedure performed by untrained or poorly trained health professionals may lead to AEs and complications that include; bleeding, injury to the penis and infection [1]. This is an indication that poor quality of service can result in medical complications and other negative health outcomes, and these will act as barriers for demand creation [15]. Healthcare service providers need to have a proper understanding of the needs and expectations of their clients, in order to deliver the best quality of services [29]. Therefore, it is important to understand the changes in quality of services after CQI support, in order to improve and strengthen the current VMMC intervention strategies.

The present study, therefore, aimed to evaluate the impact of CQI support intervention in improving quality of VMMC services across four Right to Care (RTC) supported provinces in South Africa. The RTC VMMC programme was funded to provide quality improvement support to all sites aligned with PEPFAR, NDoH and WHO standards and guidelines. The programme utilised quality improvement teams that were responsible for assessing services provided to clients, conducting support visits, providing on-site training, mentoring and assisting staff in developing quality improvement plans, standard operating procedures and tracking performance using the PDSA (Plan Do Study Act) cycle. Baseline assessments were undertaken to measure adherence to VMMC guidelines and determine the level of quality improvement support required. Continuous quality improvement support was provided to facilities based on their scores.

## Methods

### Study design

This was a pre-and post-intervention design based on routine facility level data collected as part of Right to Care quality assurance (QA) and quality improvement (QI) program for supported VMMC facilities in South Africa. VMMC facilities were assessed at baseline and after CQI support visits for continuous quality improvement from July 2018 to October 2019, in the selected districts of the North West [Dr Kenneth Kaunda, Ngaka Modiri Molema]; Mpumalanga [Nkangala]; Eastern Cape [Amathole, Alfred Nzo and O R Tambo] and Free State [Thabo Mofutsanyane] provinces of South Africa. During the period July 2018 to October 2019, 96 sites offered VMMC services [16 in the Eastern Cape, 19 in Free State, 19 in Mpumalanga and 42 in the North West province]. Out of these, 40 sites were assessed at both baseline and after CQI support visits and 13 were from Mpumalanga, 6 from Free State and 21 from the North West provinces.

### Data collection

Data were collected using a standardized VMMC CQI assessment tool developed by the National Department of Health (NDOH) and approved by PEPFAR to assess quality of services in all facilities within the districts of agreement [34]. This assessment tool has elements of the WHO-defined quality standards for VMMC service provision. The quality standard elements of focus during assessments were leadership and planning, management systems, monitoring and evaluation, group counselling, registration and communication, individual counselling and HIV testing, supplies, equipment, environment and emergency, MC surgical procedure and infection prevention.

The assessments were conducted through staff interviews, checklists and direct observation of all the facility processes and activities in line with the NDOH and WHO VMMC quality guidelines [3]. Facility level registers and client files (client intake forms, consent forms and identification documents) were also reviewed during the assessments. Quality of service assessment scores were recorded as percentages during the assessments. The scores were categorized into three groups: below 50% was an indication of “poor” service quality; 50% -80% reflected “fair” service quality; and greater than 80% indicated “good” service quality [25, 35].

### Site quality improvement support

In the present study, both internal and external activities to achieve the most desirable outcomes were employed. CQI support teams from the RTC VMMC programme were formed per district. Each CQI team was made-up of a clinical associate mentor, professional nurse mentor, enrolled nurse mentor, counsellor mentor and a data quality mentor. The teams were formed to assess services provided to clients at baseline and to conduct CQI support visits. Baseline assessments were done between July and October 2018 to identify quality of service gaps and challenges at VMMC sites using a standardized VMMC CQI assessment tool [34]. The same tool was used for the subsequent re-assessments to provide an objective and consistent measure of quality improvement. The quality standard elements of focus during assessments were as highlighted under the data collection process.

CQI support was provided to sites based on their baseline assessment scores as from December 2018 to March 2019. Sites with quality scores of less than 50% were visited quarterly for intensive CQI support. In addition, sites with 50% to 79% quality scores were visited three times annually for light CQI support, and collaborative support was provided bi-annually to sites with quality scores of 80% and above. The support provided by the CQI teams was through a combination of on-site staff training, mentorship, coaching, providing standard operating procedures and guidelines, and assistance in developing quality improvement plans. The CQI teams were also providing assistance to implement other quality improvement objectives which include tracking quality of service performance at site, district and provincial level using the Plan-Do-Study-Act (PDSA) cycle, time series charts, run charts and indicator monitoring. Routine data feedback meetings were conducted monthly to review progress against quality improvement plans developed. In addition, site improvement monitoring systems (SIMS), which is an approach to evaluate VMMC sites compliance to WHO quality standards and to determine the level of quality improvement support required was also employed during the CQI program [36].

CQI focal persons were identified at each VMMC site and these were assigned with responsibilities to focus on quality improvement requirements. Stakeholders for the VMMC program were also invited to share their success stories and barriers to program implementation as part of CQI support intervention. Following CQI implementation, post-test re-assessments were performed as from May 2019 to October 2019 to further evaluate quality of service in all supported RTC VMMC sites.

### Outcome measurement

The outcome in the present study was ‘quality of service’. It was measured at baseline and post-intervention period by checking compliance of sites to the agreed NDoH and PEPFAR quality standards based on a VMMC CQI assessment tool [34]. Mean quality of service score by province was measured as the average score across VMMC sites for each quality standard. The overall mean quality of service score was measured as the average score across all provinces for each quality standard. In addition, the overall quality of service performance score for each province was measured as the average quality of service score across all quality standards. The scores were recorded as percentages. Scores below 50% was an indication of “poor” service quality; 50%-80% reflected “fair” quality of service; and greater than 80% indicated “good” quality of service [25, 35].

### Data management and analysis

Stata version 15 (Stata Corporation, College Station, TX) was used for data management and analysis. A subset of the dataset was created for data cleaning and coding in preparation for analysis. Both original dataset and the subset of data were password protected, anonymized and stored in Google drive to maximize prevention of data loss. Paired samples t-test was used to compare the difference in quality of service mean scores between baseline assessment and after CQI support intervention by quality standard. Statistical significance was based on a p-value of less than 0.05 and the 95% confidence intervals (two-sided tests).

### Ethical clearance

Ethical clearance was obtained from the Witwatersrand Human Research Ethics Committee (HREC), South Africa [clearance number M200 415]. Permission for data access to implement the research objectives was granted by the Right to Care Not for Profit Organisation (NPO). Data was treated as confidential and the names of VMMC sites were anonymised.

## Results

### Overall quality of service by quality standard

Table 1 provides the descriptive statistics for the quality of service mean scores at baseline and after CQI support intervention disaggregated by quality standards. At baseline, the overall quality of service scores across quality standards ranged from 35±24% [leadership and planning] to 79±12 [Supplies, equipment, environment and emergency]. Similarly, after CQI support intervention, the overall quality of service scores ranged from 62±24% [leadership and planning] to 85±8% [MC surgical procedure]. The overall quality of service performance was measured as the average quality of service across all quality standards and this was 61±13% at baseline and 79±9% after CQI support intervention (Table 1).

**Table 1:** Descriptive statistics for quality of service mean scores at baseline and after CQI support intervention disaggregated by quality standards.

Province / Time point	Overall, all provinces (N = 40)		North West (N = 21)		Mpumalanga (N = = 13)		Free State (N = 6)	
	T0	T1	T0	T1	T0	T1	T0	T1
Quality Standard	Mean ± SD	Mean ± SD	Mean ± SD	Mean ± SD	Mean ± SD	Mean ± SD	Mean ± SD	Mean ± SD
Management systems	50±15	79±12	51±12	79±12	49±19	80±12	51±21	77±9
Monitoring and Evaluation	44±18	79±14	39±19	83±11	48±18	75±10	50±13	85±9
Group counselling, registration and communication	75±20	83±16	79±13	88±10	79±21	80±20	61±30	71±20
Individual counselling and HIV testing	68±25	82±18	76±14	88±9	63±33	78±21	53±33	71±29
Supplies, equipment, environment and emergency	79±12	84±9	81±12	84±10	77±13	86±7	75±1	84±5
MC surgical procedure	77±17	85±8	74±17	85±6	81±18	84±9	77±13	87±8
Infection prevention	66±18	78±12	64±14	77±10	64±18	79±18	72±19	77±7
Leadership and planning	35±24	62±24	35±27	69±25	36±17	47±16	30±27	64±19
Overall performance	61±13	79±9	63±7	81±7	61±16	76±10	59±16	76±11

NB; All values are as %

T0 = Baseline assessment, T1 = Reassessment after CQI support intervention

### Quality of service by province

The baseline quality of service scores across quality standards in the North West ranged from 35±27% [leadership and planning] to 81±12% [Supplies, equipment, environment and emergency] (Table 1). After CQI support intervention, the quality-of-service scores in the North West ranged from 69±25% [Leadership and planning] to 88±10% [Group counselling, registration and communication]. Furthermore, the overall quality of service performance score was 63±7% at baseline and 81±7% after CQI support intervention in the North West province (Table 1). In Mpumalanga and the Free State provinces, the quality-of-service scores ranged from 47±16% [leadership and planning] to 86±7% [supplies, equipment, environment and emergency] and from 64±19% [leadership and planning] to 87±8% [MC surgical procedure] after CQI support intervention respectively. In addition, the overall quality of service performance scores after CQI support intervention were 76±11% and 76±10% in the Free State and Mpumalanga provinces respectively (Table 1).

### Overall changes in quality of service by VMMC quality standard

#### Leadership and management

There was an increase after CQI support intervention in the overall quality of service for leadership and planning across provinces of 23% (95%CI: 13–34; p<0.001) (Table 2). The North West province had the highest increase of 34% (95%CI: 16–53; p<0.001) (Table 3). The increase in quality of service in the Free State province was 34% (95%CI: 9–59; p<0.05) and 11% (95%CI: 3–19, p<0.01) in Mpumalanga (Table 3).

**Table 2:** Paired samples t-test for overall changes in quality of service mean scores after CQI support intervention by quality standard across provinces.

Quality Standard indicator	Paired differences (%)	df	t-value	95% CI	Sig. (2-tailed)
Leadership and planning	23	39	28	13–34	***
Management systems	29	39	9	22–35	***
Monitoring and Evaluation	35	39	10	28–42	***
Group counselling, registration and communication	8	36	3	3–13	***
Individual counselling and HIV testing	14	36	4	7–20	***
Supplies, equipment, environment and emergency	5	39	3	1–9	**
MC surgical procedure	8	39	3	3–13	**
Infection prevention	12	39	5	7–17	***
Overall performance	18	39	10	14–21	***

*Significant, p<0.05

**Significant, p<0.01

***Significant, p<0.001

**Table 3:** Paired samples t-test (2-tailed) comparing the change in quality of service mean scores before and after CQI support intervention by province.

Quality Standard	North West					Mpumalanga					Free State				
	*T0*	*T1*	Δ	*p-value*	*95% CI*	*T0*	*T1*	Δ	*p-value*	*95% CI*	*T0*	*T1*	Δ	*p-value*	*95% CI*
Leadership and planning	35	69	34	***	16–53	36	47	11	**	3–19	30	64	34	*	9–59
Management systems	51	79	28	***	20–39	49	80	31	***	17–44	51	77	26	*	8–44
Monitoring and Evaluation	39	83	44	***	33–53	47	71	24	***	11–37	53	82	29	**	12–46
Group counselling, registration and communication	79	89	10	***	5–15	78	80	2	0.721	0–11	60	71	11	0.338	0–36
Individual counselling and HIV testing	76	88	12	**	3–20	63	78	15	*	1–29	53	71	18	0.156	9–44
Supplies, equipment, environment and emergency	81	84	3	0.291	3–8	77	86	9	*	1–17	75	84	9	*	0–19
MC surgical procedure	74	85	11	*	3–20	81	84	3	0.448	5–12	76	87	11	*	2–20
Infection prevention	64	76	12	***	5–19	64	79	15	***	9–22	71	77	6	0.530	15–26
Overall performance	62	82	20	***	15–25	61	76	15	***	8–21	59	76	17	*	6–29

*significant, p<0.05

**significant, p<0.01

***significant, p<0.001

Δ = T1 –T0, change in quality of service score, all values are as %.

T0 = Baseline mean quality score, T1 = Mean quality score after CQI support intervention

#### Management systems

Results of the paired samples t-test showed a statistically significant increase in quality of service for management systems in Mpumalanga of 31% (95%CI: 17–44; p<0.001); 28% in the North West Province (95%CI: 19–37; p<0.001) and 26% in the Free State province (95%CI: 8–44); p<0.05) (Table 3). In addition, the overall quality of service performance across provinces for management systems was 29% (95%CI: 22–35; p<0.001) after CQI support intervention (Table 2).

#### Individual counselling and HIV testing

Results showed statistically significant increase in the overall quality of service for individual counselling and HIV testing of 14% (95%CI: 7–20; p<0.001) (Table 2). The increase for individual counselling and HIV testing in the North West province was 12% (95%CI: 3–20; p<0.01) and 15% (95% CI, 1–29; p<0.05) in Mpumalanga province (Table 3).

#### Supplies, equipment, environment and emergency

There was an increase in the overall quality of service for supplies, equipment, environment and emergency across provinces and this was statistically significant 5% (95%CI: 1–9; p<0.014) (Table 2). Similarly, Mpumalanga province had an increase of 9% (95% CI: 1–17; p<0.05] and 9% (95%CI: 0–19) in the Free State province (Table 3).

#### Group counselling, registration and communication

The increase of 10% after CQI support intervention in the North West province for group counselling, registration and communication quality of service was statistically significant (95%CI: 5–15, p<0.001) (Table 3). In addition, the overall quality of service performance across provinces has significantly increased by 8% (95%CI: 3–14, p<0.001) (Table 2).

#### Monitoring and evaluation

Results of the paired samples t-test showed statistically significant increases in quality of service for monitoring and evaluation of 44% in the North West (95%CI: 34–53; p<0.001) (Table 3). Similarly, the increases in quality of service for monitoring and evaluation of 24% in Mpumalanga (95%CI: 11–37, p<0.001) and 29% in the Free State province (95%CI: 12; 46, p<0.01) after CQI support intervention were statistically significant (Table 3). In addition, the increase in overall quality of service performance across provinces of 35% for monitoring and evaluation was statistically significant (95%CI: 28; 42; p<0.001) (Table 2).

#### Male circumcision surgical procedure

There was a significant increase in quality of service after CQI support intervention for MC surgical procedure of 11% in the North West province (95%CI; 3% - 20; p<0.05) and 11% (95%CI: 2–20; p<0.05] in the Free State province (Table 3). Furthermore, the increase in overall quality of service performance across provinces of 8% was statistically significant (95%CI; 3–13; p<0.01) (Table 2).

#### Infection prevention

Infection prevention quality of service increased significantly after CQI support intervention by 12% in the North West province (95%CI: 6–19; p<0.001) (Table 3). In addition, the increase in quality of service of 15% (95%CI: 8–22, p<0.001] in Mpumalanga province was statistically significant (Table 3). Results showed a statistically significant increase in the overall quality of service performance across provinces for infection prevention of 12% (95%CI: 7–17; p<0.001) after CQI support intervention (Table 2).

#### Overall quality of service performance

Results of the paired samples t-test revealed statistically significant increase in the overall quality of service performance across provinces of 18% (95%CI: 14–21; p<0.001) after CQI support intervention (Table 2). In addition, results showed statistically significant increases for the overall quality of service performance of 15% in Mpumalanga (95%CI: 8–21; p<0.001] and 17% in the Free State province (95%CI: 6–29; p<0.05) after CQI support intervention (Table 3). Similarly, the increase in the overall quality of service the North West province of 20% (95%CI: 15–25, p < 0.001) was statistically significant (Table 3).

## Discussion

### Overall changes in quality of service

Results showed an average increase in the quality of services across provinces of 18%. The findings concur with studies that were conducted in Zimbabwe and Uganda, where positive outcomes for VMMC program were observed after CQI support intervention programs [11, 25, 36]. Furthermore, an average increase of 11% was also observed across six sites in Lesotho after CQI support [4]. Studies focusing on changes in quality of service at VMMC sites in South Africa were limited at the time this study was conducted.

### Changes in quality of service across quality standards

There were significant increases in quality of service across all quality standards from baseline to post-test assessment. Supplies, equipment, environment and emergency had the lowest change in quality of service followed by MC surgical procedure. The overall performances for the two quality standards at baseline were already close to the satisfactory category of 80% and above and this could have attributed to the low increases. This implies the majority of sites were already in compliance with the expected quality standards for supplies, equipment, environment, emergency and MC surgical procedures at baseline. CQI teams in the present study provided collaborative support for sites that were in compliance or with quality scores above 80% to maintain the satisfactory quality of service. It was also noted that intensive support through regular visits should be provided to sites with poor quality of service. Sharing of success stories and barriers to program implementation were critical to support the CQI intervention action plans. Besides the influence of other factors not assessed in the present study, the substantial increases in the overall quality of service for monitoring and evaluation, management systems, and leadership and planning were also attributed to the intensive support provided by the CQI teams. For instance, the overall quality of service for monitoring and evaluation have increased by 35% from a baseline value of 44±18%.

However, although there was a significant overall increase in quality of service score for leadership and planning, the post-test assessment mean score was lowest compared to other quality standards. This implies that more CQI support intervention programs were required to further improve quality of service for leadership and planning across all provinces. Literature shows that Kenya was successful in implementing VMMC programs as a result of strong and consistent leadership [26]. Therefore, findings from this study reflect the need to further strengthen the CQI processes for leadership and planning.

### Changes in quality of service across provinces

Quality of service scores in North West, Mpumalanga and the Free State provinces for supplies, equipment, environment and emergency were in the satisfactory category of 80% and above in the post assessment period. However, this was inconsistency with findings from previous studies where gaps were consistently identified for VMMC supplies and equipment [4, 37].

In addition, previous research shows that poor quality of service was as a result of inadequate clinical supplies and equipment [11]. The South Africa 2016 reference report on HIV and TB investment case also showed inefficiencies in the supply chain for the healthcare sector [27]. Therefore, the present study shows significant improvements across provinces in supplies, equipment, environment and emergency compared to the previous studies. Previous studies shows that mobile technology and logistics management information system (LMIS) helps to improve quality of service for the supply chain management system by creating an uninterrupted availability of material resources, medicines and equipment requirements at sites.

Although there were no significant increases in Mpumalanga for group counselling, registration, communication and MC surgical procedure, the quality-of-service scores in the post-assessment period were in compliance with the expected quality standards. The quality scores for the two standards were above 80% and this was satisfactory. Therefore, VMMC sites in Mpumalanga province were provided with collaborative support to maintain compliance across all quality standards. The Free State Province results revealed significant improvements for the majority of quality standards. However, the increases in quality of service for group counselling, registration and communication; infection prevention; and individual counselling and HIV testing did not yield statistical significance. The increases resulted in scores above 70% and this was an improvement compared to previous studies where decreases in quality of service were observed for counselling, communication and infection prevention in South Africa [4, 28–30]. Research shows that communication with clients is critical and if poorly managed can result in medical errors and increased hospital admissions [38]. Based on the finds of the present, it is important to consider the level of CQI support to be offered to VMMC sites based on assessment quality of service scores. Regular visits and intensive support are required for sites with poor quality standards.

The present study did not include the experience and level of education of the health care personnel teams that were assessed at the facilities, the effect of volume of weekly medical circumcisions and the number of CQI support visits done per facility assessed. These factors may have influenced the change in quality of service after CQI support intervention. The site readiness assessment done at the facilities could also have influenced the outcome of the baseline assessment. Furthermore, in pre and post study designs, there is a potential of one or more events, which are not part of the intervention to affect the outcome between the “before” and “after” measurements such as changes in management personnel and work processes [39]. Pre and post study designs also implies that there was no reference to a control group. Therefore, they can simultaneously generate threats to the internal validity of the research [39–41]. However, although the pre and post design is not free from limitations, it is a valid, efficient, and cost-effective way to assess program outcomes and impacts [41–43].

## Conclusions

Irrespective of the limitations of the present study, the results showed an overall increase in the quality-of-service performance across all provinces after CQI support intervention program at RTC supported VMMC sites. Collaborative support is recommended to maintain good quality of service for sites in the satisfactory category for quality of service. Regular visits and intensive CQI support are required for sites that will be performing below standards. CQI support can be provided through on-site staff training, mentorship, coaching, providing standard operating procedures and guidelines, and assistance in developing quality improvement plans.

## Supporting information

S1 Appendix Dataset. (XLS)
