# Correction: Quality of service and continuous quality improvement in voluntary medical male circumcision programme across four provinces in South Africa: Longitudinal and cross-sectional programme data

**DOI:** 10.1371/journal.pone.0302097

**Published:** 2024-04-05

**Authors:** Tawanda Nyengerai, Motshana Phohole, Nelson Igaba, Constance Wose Kinge, Elizabeth Gori, Khumbulani Moyo, Charles Chasela

The second author’s name is spelled incorrectly. The correct name is: Nelson Igaba. The fourth author’s initials also appear incorrectly in the citation. The correct citation is: Nyengerai T, Phohole M, Igaba N, Wose Kinge C, Gori E, Moyo K, et al. (2021) Quality of service and continuous quality improvement in voluntary medical male circumcision programme across four provinces in South Africa: Longitudinal and cross-sectional programme data. PLoS ONE 16(8): e0254850. https://doi.org/10.1371/journal.pone.0254850
